# Urgent surgical management for embolized occluder devices in childhood: single center experience

**DOI:** 10.1186/1749-8090-7-127

**Published:** 2012-12-07

**Authors:** Gokhan Gokaslan, Hasim Ustunsoy, Hayati Deniz, Ozerdem Ozcaliskan, Alptekin Yasim, Osman Baspinar, Gokalp Guzel

**Affiliations:** 1 Department of Cardiovascular Surgery, Gaziantep University Medical Faculty, Gaziantep, Sehitkamil, 27310, Turkey; 2 Department of Pediatric Cardiology, Gaziantep University Medical Faculty, Gaziantep, Turkey

**Keywords:** Device embolization, Septal defect, Cardiac surgery

## Abstract

**Background:**

In this study, we sought to analyze our experience in urgent surgical management for embolized cardiac septal and ductal occluder devices resulting from trans-catheter closure of atrial septal defect, ventricular septal defect and patent ductus arteriosus in childhood patient group.

**Methods:**

We retrospectively reviewed 9 patients (aged 2–15 years) who underwent urgent surgery due to cardiac septal and ductal occluder embolization between January 2007 and December 2010. Congenital defects were atrial septal defect (n = 6), ventricular septal defect (n = 1), and patent ductus arteriosus (n = 2). Risk factors for device embolization and urgent surgical management techniques for embolized device removal were discussed.

**Results:**

Removal of embolized devices in all cases and repair of damaged tricuspid valve in 2 patients were performed. Inevitably, all congenital defects were closed or ligated up to the primary defect. Total circulator arrest necessitated in 1 patient with ascending aortic device embolization. All operations were completed successfully and no hospital mortality or morbidity was encountered.

**Conclusions:**

Although closure of left to right shunting defects by percutaneous occluder devices has a lot of advantages, device embolization is still a major complication. If embolized device retrieval fails with percutaneous intervention attempts, surgical management is the only method to remove embolized devices. In this circumstance, to provide an uneventful perioperative course, urgent management strategies should be well planned.

## Background

Congenital heart diseases such as atrial septal defect (ASD), ventricular septal defect (VSD) and patent ductus arteriosus (PDA) are common type of childhood cardiac defects with an incidence of approximately 1 in 100 live births [1]. Surgical repair of all these defects was method of choice, until King et al. performed the first trans-catheter closure of ASD [2]. Reported series with percutaneous deployment of occluder devices emphasize that trans-catheter closure technique avoids open-heart surgery and its associated complications. However, these interventional procedures are accompanied with their own major complications such as device embolization. In surgical literature, management of embolized devices was discussed in case reports and recently a multicenter experience was reported [3]. There is still lack of surgical management strategies for embolized devices in childhood and in this study we aimed to share our experiences.

## Methods

The study was approved by the ethics committee of our institution, and because of its retrospective nature, patient consent was waived. This study is a review of 9 childhood patients who were urgently operated due to embolized cardiac septal and ductal occluder devices between January 2007 and December 2010 in our clinic. Preoperative and perioperative records of urgently operated 9 patients were reviewed. Each patient is defined with a number. Adult patients, patients with late device complications and uneventfully retrieved devices by percutaneous interventions were excluded.

### Statistical analysis

Data are expressed as absolute values, percentages, or mean ± SD where appropriate.

## Results

Between January 2007 and December 2010, 656 childhood patients (265 with ASD, 354 with PDA and 37 with VSD) underwent percutaneous closure of their defect at our institution. Device embolization requiring urgent surgical management occurred in 9 patients with a rate of 2.2%, 1.7% and 0.5% for ASD, VSD and PDA, respectively. Mean age was 8.8 ± 4.3 years, and six of 9 patients were female (66.6%).

Majority of the primary defects were ASD (6 patients) with a diameter equal or grater than 10 mm and closure device waist sizes were ranging 19-28mm. Mean left to right shunt ratio (Qp/Qs) in patients with ASD was 4.9 ± 3.3 (range, 1.5-11) and rim tissue around the defects was ≥ 5 mm in echocardiography before trans-catheter closure . However, aortic rim in two patients and inferior caval rim in one patient were found to be just a thin membrane in intraoperative examination. In another patient (patient number #5) who was reported to have two ASDs in echocardiography, a large single ASD divided transversely with a thin band was observed intraoperatively. In remaining two patients with ASD, device embolization was due to the learning curve of the operator.

**Figure 1:**
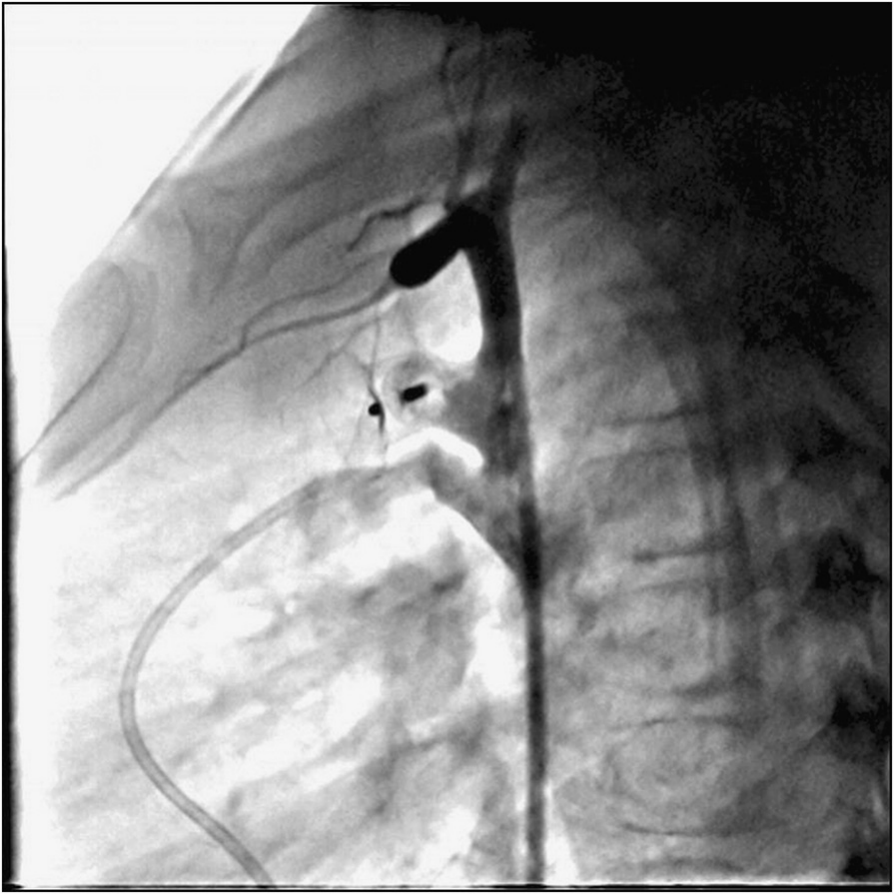
Embolised Amplatzer® ductal occluder in ascending aorta.

**Figure 2:**
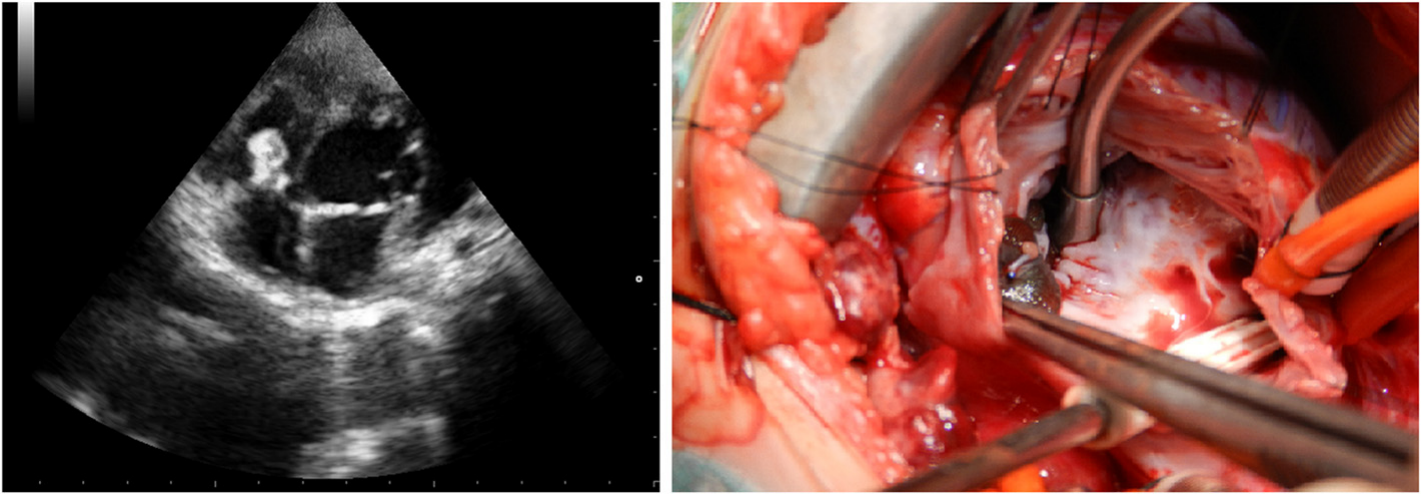
**Echocardiographic and surgical views of embolised Amplatzer® ductal occluder.** Device was tangled with tricuspid valve anterior leaflet chordas. Please note the ruptured chorda on the device which occurred during percutaneous retrieval attempts.

**Figure 3:**
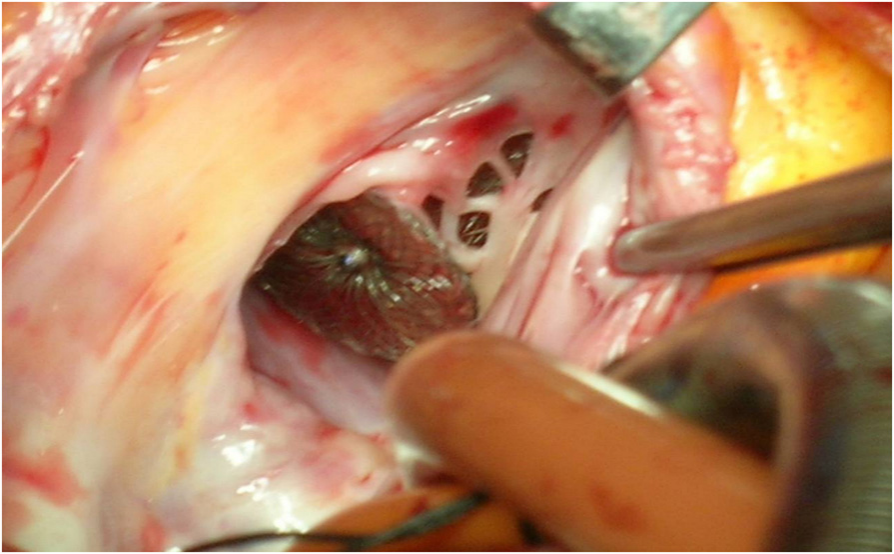
Embolised Amplatzer® septal occluder tangled with tricuspid valve and chordas.

Defects were PDA in 2 patients. Systolic gradient between aorta and pulmonary artery over PDA was 8 mmHg in patient #7 which was found low because of the concomitant ASD and 112 mmHg in patient #8 with solely PDA. Thus, the occluder device was embolized and stuck in ascending aorta in patient #7 during interventional procedure (Figure 1). Pulmonary artery embolization occurred in patient #8 just after implantation of the device and it was tangled with tricuspid valve during percutaneous retrieval (Figure 2). In patient #9 with VSD, systolic gradient between right and left ventricle was 61 mmHg. The occluder device was tangled with chordae tendineae of tricuspid valve while delivering it (Figure 3).

**Figure 4:**
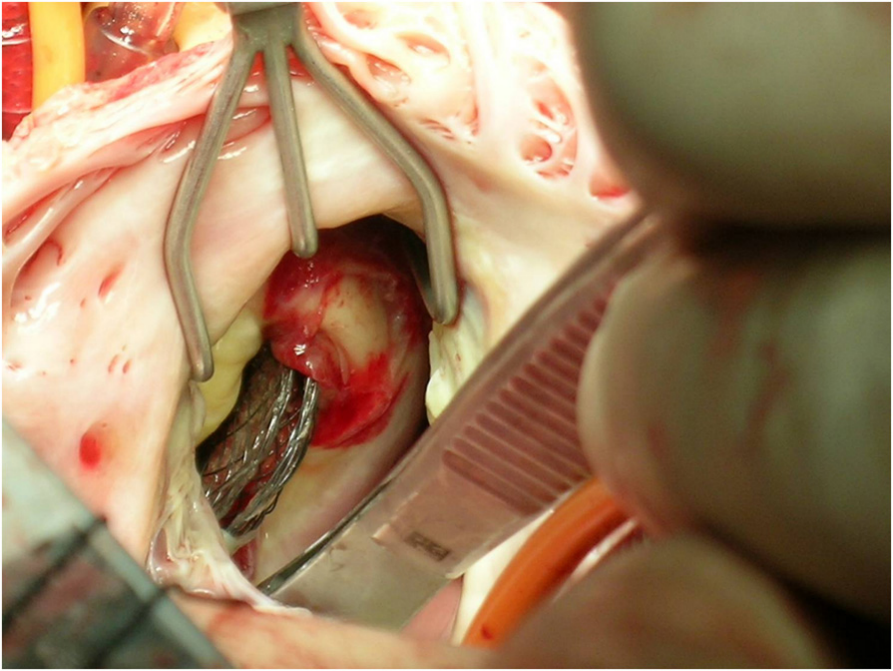
Embolised Amplatzer® septal occluder stuck in the annulus of the pulmonary valve.

Timing of events, embolization sites, embolized devices and their sizes are presented for each case in Table 1. Surgery decisions were made after failure of percutaneous attempts to retrieve embolized devices. Although all patients were heparinized during catheter intervention, six patients were additionally heparinized with 100U/kg heparin because activated coagulation time (ACT) was under 180 seconds, in order to prevent clot formation over device during surgical preparation and transfer to the operation room. Transesophageal echocardiography (TEE) was performed in all patients intraoperatively. All surgical procedures were performed through median sternotomy. Aortic cannulation, selective vena cava superior and vena cava inferior cannulations were achieved. PDA was ligated in two patients just prior to initiation of cardiopulmonary bypass (CPB). An aortic cross-clamp (CC) was applied and antegrade cold cardioplegia was administered into the aortic root to achieve prompt diastolic cardiac arrest, except one patient with ascending aortic device embolization. Snaring of both vena cava superior and inferior, right atriotomy was performed. All embolized devices were easily removed directly or by folding two opposite edges of the device via forceps even it was stuck in the annulus of the valves or fell into the left ventricle (Figure 4). Following removal of embolized devices, tricuspid septal leaflet chordae damaged in 2 patients reattached to the septal leaflet and commissure damaged in one patient was repaired with prolene suture. According to the size of the defects, ASD and VSD closures were performed directly or using a pericardial patch manner.

**Table 1:** Characteristics of patients and embolized devices

**Case No:**	**Age (Years)**	**Primary disease**	**BSA (m**^**2**^**)**	**Defect size (mm)**	**Qp/Qs**	**Embolized device and size (mm)**	**Embolization site**	**Timing of event**
**1**	4	ASD	0.66	20	6.08	Amplatzer® 9-PFO-024(38–34)	Tricuspid valve	1 day
**2**	8	ASD	0.84	19	5	Amplatzer® 9-PFO-022(32–36)	Right ventricle	1 day
**3**	9	ASD	0.9	22	11	Amplatzer® 9-PFO-026(36–40)	Left ventricle	Peroperative
**4**	15	ASD	1.26	27	3.5	Amplatzer® 9-PFO-019(29–33)	Pulmonary valve	1 day
**5**	15	ASD	1.57	27-28	4	Amplatzer® 9-PFO-028(38–42)	Right atrium	Peroperative
**6**	9	ASD	1.08	10	1.5	Biostar BSR-28	Tricuspid valve	2 days
**7**	8	PDA	0.81	4.5	1.4	Amplatzer® 9-PDA006(10–8)	Ascending Aorta	Peroperative
**8**	2	PDA	0.45	4.55	3.1	Amplatzer® Nit.Ocluder(9–6)	Tricuspid valve	Peroperative
**9**	10	mVSD	0.79	7	2.2	Amplatzer® 9-VSDmusc-008(16)	Tricuspid Valve	Peroperative

*ASD*: Atrial septal defect, mVSD: Muscular ventricular septal defect, *PDA*: Patent ductus arteriosus, *Qp/Qs*: Left to right shunt ratio.

In patient with ascendant aortic embolization (patient #7 with PDA and concomitant ASD), device position was confirmed with TEE following initiation of CPB and patient was cooled to 20 degree Celsius. Thereafter distal ascending aortotomy was performed just left lateral side of the aortic cannula via total circulator arrest (TCA) of 6 minutes. Embolized device was removed and aortotomy was closed in two layers. While heating the patient, direct closure of additional secundum ASD was performed through the right atriotomy. In early postoperative echocardiography, systolic pulmonary artery pressure was shown decreased from 82 to 45 mmHg. Surgical management data are presented for each patient in Table 2.

**Table 2:** Surgical management data

**Case No:**	**Age (Years)**	**Primary disease**	**TCA**	**CC**	**CPB**	**Surgical management**
			**(Minute)**	**(Minute)**	**(Minute)**	
**1**	4	ASD	-	28	44	ASO removal and ASD pr.
**2**	8	ASD	-	37	45	ASO removal and ASD rpp.
**3**	9	ASD	-	18	30	ASO removal and ASD pr.
**4**	15	ASD	-	30	45	ASO removal and ASD rpp.
**5**	15	ASD	-	28	37	ASO removal and ASD rpp.
**6**	9	ASD	-	33	42	ASO removal, Tricuspid valve septal leaflet chorda and posterior leaflet septal commissure repair, ASD rpp
**7**	8	PDA	6	0	116	PDA ligation, Coil removal via aortotomy with TCA and ASD pr.
**8**	2	PDA	-	30	42	PDA ligation, Tricuspid valve septal leaflet corda repair
**9**	10	mVSD	-	182	214	VSO removal, 2 small muscular and 1 cm inlet VSD rpp
Mean				42.8 ± 53.3	68.3 ± 60.2	

*ASD*: Atrial septal defect, *ASO*: Atrial septal occluder, *CC*: Cross clamp time, *CPB*: Total cardiopulmonary bypass time, *mVSD*: Muscular ventricular septal defect, *PDA*: Patent ductus arteriosus, pr: Primer repair, *Qp/Qs*: Mean left to right shunt ratio, rpp: repair with pericardial patch, *TCA*: Total circulatory arrest time, *VSO*: Ventricle septal occluder.

The mean time interval between decision of the surgical removal of the embolised device and surgical treatment was 65 ± 16.5 (range, 40–90) minutes. Mean blood transfusion was 1.88 ± 0.64 units. Mean duration of intensive care unite stay was 1.2 ± 0.44 days and mean total hospital stay was 4.9 ± 1.3 days. Any tromboembolism or peripheral artery complication including ischemia was not observed postoperatively. No mortality or surgical complication was encountered during hospital stay. The mean follow-up period was 37.8 ± 17.1 months. All patients were free of cardiac symptoms and there was no residual defect in their one-year and last follow-up. In two patients with tricuspid valve repair, mild regurgitation was reported in their last follow up echocardiography.

## Discussion

Interventional trans-catheter closure for all types of cardiac defects with left to right shunting gained wide popularity and became a standard technique against surgery for nearly two decades all over the world. Recent studies including both adult and childhood patients emphasizes that trans-catheter closure is a safe method with shorter hospital stay, less mortality and morbidity rates. Moreover, it also obviates surgical risk factors and inevitable operation scar of surgery [4-7]. However, this procedure is not free of complications. Embolization of the device may occur in unexpected sites of circulatory system and may cause serious damages. In the literature, embolization rates were reported 4% in 1991 [8], 20% in 1996 [9], and dramatically decreased to 0.55% in 2005 with new generation devices [10]. In a review reported in 1996, suggested mechanisms for acute failure were operator related factors resulting from inadequate experience (learning curve), inaccurate deployment, inadequate defect rim to hold the device, tearing of the interatrial septum especially at the lower rim of the ASD owing to catheter and device manipulations and Sideris buttoned device itself [11]. Berdat et al. reviewed their surgical experiences with embolized Sideris and ASDOS devices and suggested that new generation Amplatzer® devices indicated more promising results [12]. Despite new generation occluder devices were developed, embolized devices which require surgical management are still reported and majority of these are case reports [13-15]. In a recent multi-center study which investigates device embolization complications, 3 patients died of stroke due to cerebral embolism and cardiac perforation was noted in one patient. The authors concluded that ‘Once a complication leading to surgery occurs, mortality is significantly greater than that of primary surgical ASD closure’ [3].

In our study majority of primary defects were ASD and embolization rate was 2.2%. Patient selection for ASD closure with occluder device is generally performed according to the sizes of the defect and rim. Misra et al. reported their experience with defective aortic rim which caused device embolization to pulmonary artery and they emphasized the importance of the aortic rim in device deployement [16]. If rim thickness is weak or just a membrane, this may lead device embolization. In our study, aortic rim in two patients and inferior cava rim in one patient were a thin membrane. Additionally, in patient # 5 who was thought to have two ASDs in echocardiography, intraoperative investigation revealed that there existed actually one large ASD divided by a thin band. Combination of thin rim and high Qp/Qs might have been responsible for the embolization in these patients. Moreover, during the percutaneous retrieval attempts, intracardiac structure damage may occur particularly at valvular and subvalvular apparatus. Within our experience, tricuspid septal leaflet chordae injury in two cases and posterior leaflet septal commissure damage in one case were encountered during operations. These injuries were repaired successfully.

Device embolization rate during trans-catheter PDA occlusion was reported as 0.5% in a multi-center study [17]. Successful surgical removal of embolized PDA devices from pulmonary arteries and descending aorta were reported formerly [13,14]. Magee et al. suggests that embolization of the PDA device more commonly occurs in the pulmonary circulation than the systemic circulation because of the pressure gradient [18]. We encountered with embolized PDA devices in two cases (patient #7 and #8). PDA diameters were 4.5 and 4.55 mm. Although ductal diameters were small, gradients between aorta and pulmonary artery were the reason for the device embolization. In patient #7 with concomitant ASD, PDA occluder device dislodged and migrated to ascending aorta due to high pulmonary pressure. In patient #8, gradient was 112 mmHg and device was embolized to pulmonary artery. Although PDA diameters were similar, the gradient in these cases determined the dislodgement of the device and the embolization site.

Holzer et al. reported device embolization rate of 2.7% in muscular VSD closure in both adult and childhood patients [19]. In 2007, device embolization rate was reported as 0.9% for the European registry of Amplatzer® VSD occluder [6]. In a review reported in 2010, the author emphasizes TEE guidance in trans-catheter device closure of muscular VSD [20]. In our data, device embolization occurred in one patient (patient #9) with muscular VSD. Device was tangled with tricuspid valve chordae during deployment probably because of the lack of TEE guidance.

Device removal was performed through the right atrium in our 8/9 patients. Performing right atriotomy provided us a good surgical exposure to reach all intracardiac structures even to the left ventricle via ASD. Devices even stuck to the valvular annulus were removed easily via folding their edges with forceps. Intracardiac structure damages which possibly occurred due to percutaneous device retrieving attempts at valvular and subvalvular apparatus were repaired successfully. Only one of the devices which was embolized to the ascending aorta was removed via TCA after confirming its position with TEE. Perioperative TEE guidance is essential particularly in these patients, because the device migration might occur after aortic cannulation and initiation of CPB [15].

**Figure 5:**
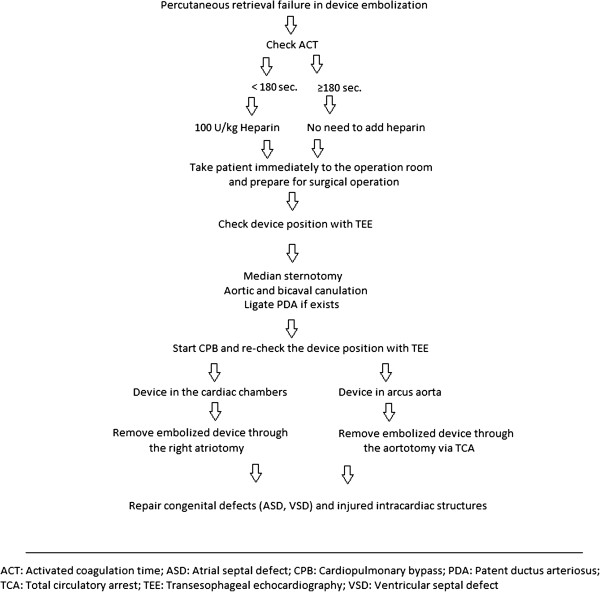
A possible algorithm for surgical management of complicated embolized devices.

In post operative period, fortunately we did not encounter any embolism, tamponade or arrhythmias. The reasons for no death or uneventful course in our operations may be related to stand-by cardiac surgical team, additional heparinization of the patients after the onset of embolization, appropriate surgical management strategy. An algorithm may be suggested considering our limited experiences together with the results of the literature for a more helpful surgical management strategy. A possible algorithm is indicated in Figure 5.

## Conclusions

Occluding of left to right shunting defects by devices is less invasive than surgery. Echocardiographic evaluation of rim quality and measurement of the gradients may help choose the appropriate patients for interventional procedures. During the intervention, TEE guidance is needed to confirm the echocardiographic findings and support the deployment procedures. Despite all, embolization is still a major complication in interventional closure of the defects. If percutaneous catheter retrieval attempts fail, surgical management is the only method to remove the embolized devices. However, an urgent surgery in a patient who already had many hours of anesthesia perhaps with compromised hemodynamics due to blood loss and/or damaged intra cardiac structures is supposed to have more intraoperative or postoperative complications than an elective surgical repair of the primary lesion. Our experiences without any serious complication may be attributed to the appropriate surgical management strategy.

## Competing interests

The authors declare that they have no competing interests.

## Authors’ contributions

GG and UH carried out the study design and drafted the manuscript, DH, OO and GG collected patients’ data, YA and BO participated in the design of the study. All authors read and approved the final manuscript.
